# Non-ST Elevation Myocardial Infraction after High Dose Intravenous Immunoglobulin Infusion

**DOI:** 10.1155/2009/861370

**Published:** 2010-02-22

**Authors:** Meir Mizrahi, Tomer Adar, Efrat Orenbuch-Harroch, Yair Elitzur

**Affiliations:** ^1^Internal Medicine A, Department of Medicine, Hebrew University-Hadassah Medical Center, Jerusalem IL 91120, Israel; ^2^Cardiology Department, Hebrew University-Hadassah Medical Center, Jerusalem IL 91120, Israel

## Abstract

Intravenous immunoglobulins (IVIgs) are used for several indications, including autoimmune conditions. IVIg treatment is associated with several possible adverse reactions including induction of a hypercoagulable state. We report a 76-year-old woman treated with IVIg for myasthenia gravis, which developed chest pain and weakness following IVIg infusion. The symptoms were associated with ST segment depression in V4–6 and elevated troponin levels. The patient was diagnosed with non-ST elevation myocardial infarction (NSTEMI). The patient had no significant risk factor besides age and a cardiac perfusion scan was interpreted as normal (the patient refused to undergo cardiac catheterization). This case is compatible with IVIg-induced hypercoagulability resulting in NSTEMI. Cardiac evaluation should therefore be considered prior to initiation of IVIg treatment especially in patients with multiple cardiovascular risks.

## 1. Introduction

Intravenous immune globulin (IVIg) is a solution of human plasma-derived immunoglobulins of over a thousand donors containing an extensive range of immune antibodies which may serve in protecting against human pathogens and foreign antigens. The mode of action of IVIg is complex and involves several mechanisms that act in synergy [1]. The therapeutic effects of IVIg most likely reflect the functions of natural antibodies in maintaining immune homeostasis in healthy individuals. Different doses of IVIg are used for different diseases, for example, in immunodeficit disease the preferred dose is 200–400 mg/kg body weight, given approximately every 3 weeks. On the other hand, “high doses” of IVIg, 1-2 g/kg, are used as an “immunomodulatory” agent in autoimmune and inflammatory disorders [1]. Its capacity to exert a variety of immunomodulating activities has led to the growing use of IVIg in treating several immune-mediated disorders and autoimmune diseases such as systemic lupus erytematous (SLE), antiphospholipid syndrome (APS), pemphigus, idiopathic thrombocytopenic purpura (ITP), multiple sclerosis (MS), myasthenia gravis (MG), Kawasaki syndrome, dermatomyositis (DM) polymyositis (PM), juvenile dermatomyositis (JDM), systemic vasculitides, adult Still's disease, prevention of graft-versus-host disease in recipients of allogeneic bone marrow transplants, intestinal bleeding due to Henoch-Schonlein purpura and in recurrent abortions [2–10]. 

The majorities of these adverse effects attributed to IVIg are mild, self-limited, and related to the speed of infusion. These effects include headache (50%), back pain (4–6%), chills, myalgia (4%), cough (2%), fever (1%), or chest discomfort and do not usually necessitate discontinuation of therapy. Severe adverse reactions occur with an incidence of <5% and include aseptic meningitis, dermatologic reactions, anaphylaxis, and renal tubular necrosis in patients with pre-existing kidney disease and volume depletion [11, 12]. 

Although an association between IVIg administration and myocardial infarction (MI) has not been yet established in prospective clinical trials, clinical experience suggests that elder individuals or those with ischemic heart disease are potentially at risk for cardiac ischemia with IVIg administration [13, 14].

We report a case of probable IVIg-induced acute MI occurring during treatment for myasthenia gravis.

## 2. Patient Description

A 76-year-old woman was admitted to the emergency room (E.R) due to loss of consciousness (syncope) 2 hours following IVIg administration. Her past medical history included hypothyroidism, gastroesophageal reflux, right lumpectomy, and myasthenia gravis (MG) which was diagnosed 5 months earlier. The chronic medical treatment of the patient was brotizolam 0.25 mg/once daily, lorazepam 1 mg/once daily, simvastatin 20 mg/once daily, thyroxine sodium 100 mg/once daily, amlodipine 5 mg/once daily, acetyl salicylic acid 75 mg/once daily, pyridostigmine Bromi 60 mg/3 times a day. After an MG diagnosis was made, physostigmine treatment was initiated with partial response, after which additional treatment was given with azathioprine 100 mg/day. This was discontinued due to diverticulosis, and the patient started treatment with 2 mg/kg of IVIg once monthly (Gamimune—Igs normal Human 30%). The patient was admitted on the first day of her 3th cycle of IVIg treatment. 

Anamnesis revealed that when the IVIg infusion ended, the patient felt extremely weak with dizziness and chest pain. The patient denies any history of chest pain or cardiac catheterization, smoking, hyperlipidemia, diabetes, or a family history of cardiac disease.

**Figure 1:**
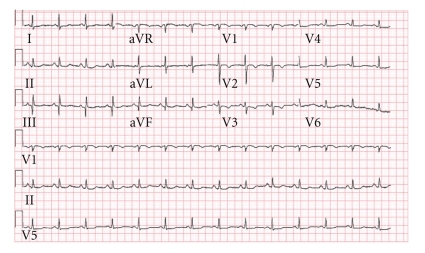


On arrival to the E.R, her vital signs showed slight orthostatic blood pressure with 113/80 mm/Hg in the supine position and 98/75 mm/Hg in the upright position, heart rate was 99 bpm, the rest of her physical examination was unremarkable; electrocardiogram (ECG) showed ST depression and T wave inversion in the lateral (V4–V6) and anterior wall (V2-3), which were not demonstrated on a prior electrocardiogram examination (Figure 1). Blood tests showed normal electrolytes levels with sodium levels of 139 mmol/L (Normal range 135–145 mmol/L), potassium levels of 3.6 mmol/L (Normal range 3.5–5 mmol/L), and magnesium levels were 0.9 mmol/L (Normal range 0.7–0.95 mmol/L); renal function was unremarkable with creatinine levels of 86 *μ*mol/L (Normal range 60–106 *μ*mol/L) and urea levels of 5.2 mmol/L (Normal range 3.3–6.5 mmol/L), liver function tests were in the normal range with ALT levels of 31 units/L (Normal range 6–53 units/L), AST levels of 58 units/L (Normal range 2–60 units/L), ALK.P levels of 69 units/L (Normal range 40–130 units/L), GGTP levels of 16 units/L (Normal range 10–80 units/L), and LDH levels of 520 units/L (Normal range 300–620 units/L). Complete blood count showed leukocytosis of 15.1 10E9/l (Normal range 4–10 10E9/l), with 88% neutrophilis, thrombocytopenia of 111 10E9/l (Normal range 140–400 10E9/l), hemoglobin (Hb) level on patient's arrival was 15.1 g/dL (Normal range 12–15 g/dL) when the patient base line levels are 12 g/dL and hematocrit (Hct) levels of 42.4 (Normal range 38–52%). Erythrocyte sedimentation rate (ESR) was 45 mm/h, C-reactive protein (CRP) was 2.3 (N-0.5). Cardiac markers showed elevated troponin T levels of 0.331 ng/mL (Normal range 0-0.1 ng/mL) and on repeated testing after six hours, a rise in troponin T levels was seen up to 0.478 ng/mL.

The patient was admitted to the cardiology department with a diagnosis of non-ST elevation MI (NSTEMI) and was treated with full anticoagulation—enoxaparin of 60 mg twice a day and clopidogrel 75 mg/day (after a 300 mg loading dose), *β*-blockers such as metoprolol 12.5 mg twice daily, ACE-I as ramipril 2.5 mg once daily and acetyl salicylic acid 100 mg/day. 

During hospitalization the patient was offered cardiac catheterization, which she refused. She underwent a thallium cardiac scan which was interpreted as normal. In light of normal thallium cardiac scan and a stable clinical course, the patient was discharged after 6 days and a follow-up in the cardiology clinic was recommended. Ten days after the initial event the patient underwent cardiac tomography with no evidence of coronary disease and calcium index was in the normal range.

## 3. Discussion

We report here a rare case of non-ST elevation myocardial infraction after IVIg treatment. Although several case reports of myocardial infraction after the use of IVIg were published, it is not generally considered an adverse effect of IVIg [13–15]. Rapid administration of IVIg may cause flushing, altered heart rate, blood pressure. Medical literature showed a very low rate of venues thromboembolic events in young patients with MS treated with low-rate infusion [16]. The recommended initial infusion rate is 0.5 mL/kg/h for a 5% IVIg solution and may be titrated up to 4 mL/kg/h as tolerated [16].

The pathophysiology of IVIg-induced thrombosis is not well recognized. Proposed mechanisms consist of platelet or endothelial cell activation and increased blood viscosity, which is a significant determinant of subendocardial oxygen delivery [17, 18]. Other proposed mechanisms include increased release of vasoconstrictive cytokines and/or arterial vasospasm caused by IVIg, which can contribute to thrombotic events [19], in patient with Guillain-Barré Syndrome, specially in adult patient the use of IVIg can provoke Reversible posterior leukoencephalopathy, cerebral vasoconstriction, and strokes; cases in pediatric patients were described also in [20, 21]. Dalakas et al. reported an increase in viscosity after IVIg from 0.1 to 1 centipoise in 13 patients, with the increase being higher in patients with paraproteinaemic polyneuropathy and recommended monitoring of serum viscosity in elderly patients and in those with paraproteinaemias, high lipoproteins values, or pre-existing vascular diseases. On the other hand slow infusion rate and young age are optimal conditions for low risk of VTE post-IVIg infusion [15–19].

We recognize that our patient may have had characteristics complicating the clarity of a direct relation between IVIg administration and acute MI (a direct causal relationship between IVIg administration and myocardial infarction was not proved with certatinty). Nevertheless, the fact that our patient was free of prior ischemic heart disease, with a normal Thallium scan and a negative cardiac tomography test result, after the initial event, increases the likelihood of a direct connection between higher blood viscosity due to IVIg administration and the cardiac event. Therefore, suggesting a temporary ischemic mechanism, possibly a transient increase in viscosity or reactive vasospasm similar to the mechanism which provokes posterior leukoencephalopathy and vasoconstriction in Guillain-Barré Syndrome patients treated with IVIg. Further treatment with IVIg with higher rate of hydration and slower rate of drug administration did not reproduce this ischemic episode in our patient.

## 4. Conclusion

Frank coronary events due to IVIg administration are still considered rare. It is difficult to ascertain the true incidence of MI and other thrombotic complications of this treatment, as many similar cases remain unpublished and those cases which are reported are highly variable in the details provided, therefore limiting informed conclusions. Cardiovascular evaluation is not routinely performed before IVIg treatment; however, it is recommended to be taken into consideration in elderly patients with multiple risk factors for cardiovascular disease who are candidates for IVIg treatment according to the published recommended rate and dosage. Furthermore in those patients the cardiac monitoring during the IVIg treatment needs to be considered.

## Abbreviations

IVIg: Intravenous immunoglobulin
NSTEMI: Non-ST elevation
ER: Emergency room
ECG: Electrocardiogram
MG: Myasthenia gravis.
